# Clinical features and treatment of stroke-like episodes in mitochondrial disease: a cohort-based study

**DOI:** 10.1007/s00415-024-12745-y

**Published:** 2024-12-12

**Authors:** Nora Mickelsson, Jussi Hirvonen, Mika H. Martikainen

**Affiliations:** 1 Clinical Neurosciences, Department of Clinical Medicine, University of Turku, Turku, Finland; 2 Neurocenter, Turku University Hospital, Turku, Finland; 3 Department of Radiology, Turku University Hospital, Turku, Finland; 4 Department of Radiology, Faculty of Medicine and Health Technology, University of Tampere, Tampere, Finland; 5 Research Unit of Clinical Neuroscience, Neurology, University of Oulu, Oulu, Finland; 6 Neurocenter and Medical Research Center, Oulu University Hospital, Oulu, Finland; 7 Present Address: Faculty of Medicine, University of Oulu, Aapistie 5A, 90220 Oulu, Finland

**Keywords:** Genetics, Epilepsy, Mitochondrial diseases, Mitochondrial DNA, Stroke-like episode

## Abstract

**Background:**

Stroke-like episode (SLE) is a subacute evolving brain syndrome in patients with primary mitochondrial diseases. Despite previous research, the understanding of the clinical spectrum, treatment, and outcomes of mitochondrial SLEs is far from complete. In this single centre study, we report the clinical symptoms and radiological findings as well as the medical treatment and outcomes of SLEs in patients with mitochondrial disease.

**Methods:**

This retrospective, observational study during years 2000–2023 was based on a cohort of patients diagnosed with mitochondrial disease at Turku University Hospital (TUH; Turku, Finland) in the region of Southwest Finland. Data were obtained from the hospital electronic medical record system.

**Results:**

The investigated cohort consisted of 76 patients (37 men, 39 women) with a diagnosis of mitochondrial disease. Among these, 12 patients had a history of at least one SLE; the total number of SLEs was 20. The most common genetic aetiology among patients with SLEs was m.3243A > G (N = 7). The mean age at first SLE was 40 years (range: 5–66 years), and the mean interval between episodes was 4.8 years (range: 4 months—10 years). The duration of episodes varied between 1 and 193 days (median 14 days, mean 37 days); 10 patients needed intensive care unit (ICU) treatment. The mean survival time between the first SLE and death was 3.6 years (range: 0–16 years).

**Conclusion:**

Our study highlights the importance of early recognition and prompt management of SLE symptoms, especially epileptic seizures, in this life-threatening entity.

## Introduction

Mitochondrial diseases are inherited neurometabolic diseases that affect mitochondrial oxidative phosphorylation. They are caused by mutations in either mitochondrial DNA (mtDNA) or nuclear DNA (nDNA), and the prevalence is estimated to be between 1:4000–1:5000 [1–3]. The considerable variability of clinical symptoms associated with mitochondrial disease remains a major diagnostic challenge for clinicians.

Stroke-like episodes (SLEs) have first been described in mitochondrial syndromes of mitochondrial encephalomyopathy, lactic acidosis, and stroke-like episodes (MELAS), most commonly associated with the m.3243A > G variant in mtDNA [4, 5]. The m.3243A > G variant is indeed the most common aetiology of SLE, but other pathogenic mitochondrial DNA variants and nuclear gene mutations are also recognized in association with SLEs [6–8]. The clinical features of SLE typically include headache, nausea or vomiting, encephalopathy, seizures, visual disturbances, language or other cognitive impairments, and sensory-motor symptoms [9]. The onset is typically subacute or acute, and the associated stroke-like lesions observed with brain imaging evolve over time. The distribution of the lesions is inconsistent with a vascular territory, and they are commonly located in posterior brain regions [10, 11]. SLEs were long thought to be associated only with mitochondrial disease at younger ages (< 40 years), but late-onset presentations are increasingly recognized [6].

The outcome of SLE varies among patients. The neurological symptoms and lesions can spontaneously reverse, but especially patients with mitochondrial disease related to pathogenic variants in the nuclear gene *POLG* are at higher risk of death from status epilepticus [12]. Indeed, the recent consensus-based report on the definition and management of SLE highlights the importance of rapid antiepileptic treatment [9].

Despite previous research, the understanding of the clinical spectrum, treatment, and outcomes of mitochondrial SLEs is far from complete. We systematically investigated the clinical symptoms and radiological findings as well as the medical treatment and outcomes of mitochondrial disease patients with SLEs in a single centre mitochondrial disease patient cohort.

## Materials and methods

This retrospective, observational study was based on a cohort of mostly adult patients diagnosed with mitochondrial disease at Turku University Hospital (TUH; Turku, Finland) in the region of Southwest Finland. Most of these mitochondrial disease patients had regular follow-ups at TUH. Patient data were obtained from the TUH electronic medical record system (earliest available date January 1, 2000).

We included all patients with a diagnosis of mitochondrial disease who had at least one SLE in their medical history. SLEs were confirmed by scrutinizing patient medical notes and relevant brain imaging investigations. A stroke-like episode was defined as a clinical event with new focal neurological dysfunction, bilateral convulsion, altered consciousness, or any combination of these features, with concurring, anatomically relevant acute (or subacute) cortical and subcortical abnormalities in brain imaging [6]. We also included SLEs in adult patients with clinical episodes concurrent with SLE and abnormal EEG finding but no acute stroke-like lesion in brain CT, and clinically diagnosed SLEs in paediatric patients with m.3243A > G and MELAS with previous SLE confirmed by brain imaging. The medical notes related to SLEs were systematically reviewed, including initial symptoms, medical treatments, and clinical outcomes related to SLEs, as well as brain imaging data. The data collection was completed in 2022–2023.

This research was covered by the TUH research permission TO4/016/16. Individual informed consent was not required for this retrospective, register-based study.

## Results

The initial study population consisted of 76 patients (37 men, 39 women) with a diagnosis of mitochondrial disease. Of these, 12 patients (16%) had a history of at least one SLE, the total number of episodes being 20 (Table 1). All patients were of Caucasian (Finnish) origin.

**Table 1:** Mitochondrial disease patients with a history of stroke-like episodes

Patient	Current age or age at death (y)	Sex (F/M)	Molecular diagnosis	MD symptoms before first SLE	Age at SLE (y)	Number of SLEs
P1	59^†^	M	m.3243A > G	DM, SNHL, recurrent pancreatitis	55, 59	2
P2	66^†^	F	m.3243A > G	DM, SNHL	66	1
P3	11	M	m.3243A > G	Developmental delay	5	1
P4	56	F	m.3243A > G	SNHL, cardiomyopathy, gastrointestinal symptoms	55	1
P5	26	F	m.3243A > G	Developmental disability, microcephalia	10, 15, 22, 25	4
P6	66	F	m.3243A > G	DM, SNHL, migrainous headaches	51	1
P7	55	F	m.3243A > G	DM, sensorineural hearing loss, cardiomyopathy	54	1
P8	30^†^	F	W748S homozygous variant in *POLG*	Tremor, vertigo, fatigue, exercise intolerance	26, 29	2
P9	61^†^	M	W748S homozygous variant in *POLG*	Epilepsy, ataxia, dysarthria, peripheral neuropathy, cognitive decline	61	1
P10	20^†^	F	W748S homozygous variant in *POLG*	Epilepsy, migrainous headaches	20	1
P11	66^†^	F	m.3271T > C	Sensorineural hearing loss, migrainous headaches	65, 66	2
P12	31^†^	M	m.8344A>G	Myoclonic epilepsy, ataxia, peripheral neuropathy, cognitive decline, migrainous headaches	15, 25, 31	3

*DM* diabetes mellitus, *F* female, *M* male, *MD* mitochondrial disease, *SLE* stroke-like episode, *SNHL* sensorineural hearing loss, † deceased.

The most common genetic aetiology among patients with SLEs was m.3243A > G (N = 7); others included the W748S variant in *POLG* (N = 3), m.3271 T > C (N = 1) and m.8344A > G (N = 1). From the overall study population (N = 76) 28 patients (37%) harboured m.3243A > G. Of the 12 patients with SLEs, five were alive at the time of data collection. Among the deceased, the mean age of death was 48 years (range: 20–66 years) in the SLE group and 51 years among those mitochondrial patients who had no SLE in their medical history (8 patients).

Five out of the 12 patients had more than one SLE (1–4 episodes per patient). The mean age at first SLE was 40 years (range: 5–66 years) and the mean time between episodes was 4.8 years (range: 4 months—10 years). Two of the m.3243A > G patients had their first SLE before the age 11, but others later, at ages of 51–66 years. The two children manifested developmental delay before their first SLE; this was however not observed among patients who had their first SLE in adult age.

All the patients included in this study were admitted to the hospital during their stroke-like episodes. In six cases, SLE symptoms began on the same day the patient was admitted to the hospital. In 14 cases, the symptoms started 1–16 days before (mean: 4.2 days). The mean hospital stay was 30 days (range: 1–113 days). In 10 episodes, patients were also admitted to the ICU, the mean time being 15 days (range: 1–84 days) (Table 2). SLE was the main cause of death in 7 patients. The mean survival time between the first SLE and death was 3.6 years (range: 0–16 years).

**Table 2:** Symptoms, findings, and treatment related to stroke-like episodes

Patient	SLE #	Main symptoms	ICU (d)	Hospital (d)	ASMs used	Anesthetic	Plasma lactate (mmol/l)	Brain imaging	EEG
**P1**	1	Altered conscious level, generalised convulsive seizures	3	15	Levetiracetam, benzodiazepines (lorazepam, midazolam, diazepam)	Propofol	2.1	Cortical edema with restricted diffusion in right temporo-parietal region Small subdural hematoma (MRI)	Periodic slow wave focus in right frontal area Mild generalized slowing No epileptic discharges
	2	Confusion, altered conscious level, focal motor seizures	6	6	Levetiracetam, lacosamide, perampanel, lorazepam	Propofol, ketamine	1	Large SLL in the left occipito-parietal area (CT)	Persistent seizure activity in the left hemisphere, corresponding to focal NCSE
**P2**	3	Altered conscious level, generalised convulsive seizures, paralytic ileus, focal motor weakness	8	8	Levetiracetam	Propofol	3.4	Artifacts from right sided cochlear implant Central, cortical, and cerebellar atrophy (CT)	Severe generalized slowing Interictal focal epileptic discharges in the posterior regions of right hemisphere
**P3**	4	Nausea and vomiting, headache, focal motor weakness, altered conscious level, dysphasia	7	21	No ASM	Propofol	4.8	Acute SLL in the left basal ganglia and insular cortex area (MRI)	N/A
**P4**	5	Visual hallucination, nausea, altered conscious level, generalized convulsive seizures	2	10	Levetiracetam, lacosamide, diazepam	Propofol	8	Basal ganglia calcification, moderate white matter lesions (CT)	Mild generalized slowing No epileptic discharges
**P5**	6	Nausea and vomiting, focal and generalized convulsive seizures	2	3	Fosphenytoin, oxcarbazepine, barbiturate, diazepam	Propofol, thiopental	N/A	Large SLL in the right parietal area, cortex involved, showing restricted diffusion Small lesions in the right parietal and occipital lobes and both sides on temporal lobe Small lesion in the left insular cortex (MRI)	Focal slowing in the posterior regions of the right hemisphere No epileptic discharges
	7	Focal motor seizures, nausea and vomiting, focal motor weakness, dysphasia	0	1	Lamotrigine (home dose raised), diazepam		3.2	N/A	N/A
	8	Nausea, headache, constipation, fatigue, dehydration	6	6	No changes in ASM	–	2.1	N/A	N/A
	9	Nausea, vomiting, constipation, slight fever, dehydration	2	6	No changes in ASM	–	4.8	N/A	N/A
**P6**	10	Focal motor weakness, headache	0	15	No ASM	–	1	Small SLL in the left thalamus area (MRI)	N/A
**P7**	11	Confusion, visual field defect, dysphasia, headache, focal motor weakness	0	47	Levetiracetam	–	2.3	Acute SLL in the left temporo-parieto- occipital area Severe cerebellar atrophy (MRI)	Focal slowing in the left temporo-occipital region Mild generalized slowing
**P8**	12	Headache, nausea, visual hallucinations, visual field defect, dysphasia, generalised convulsive seizures	0	11	Levetiracetam, fosphenytoin, oxcarbazepine, diazepam	–	N/A	SLL in the left parieto-occipital area (MRI)	Interictal focal epileptic discharges in the left temporo—occipital region
	13	Headache, visual hallucination, generalized convulsive seizures, slight fever, focal motor weakness	33	98	Levetiracetam, lacosamide, fosphenytoin, topiramate, gabapentin, barbiturate, benzodiazepines (midazolam, diazepam)	Propofol, thiopental, midazolam, ketogenic diet	0.8	SLL in the right putamen, caudate nucleus, and thalamus -Show T2 hyperintensity and restricted diffusion (MRI)	Interictal PLD´s in the posterior regions of the right hemisphere
**P9**	14	Generalized convulsive seizures	0	17	Levetiracetam, gabapentin (home dose raised), carbamazepine (regular medication), benzodiazepines (diazepam, lorazepam)	–	N/A	Central, cortical, and cerebellar atrophy Severe white matter lesions in cerebellum and brainstem (CT)	Generalized SE finding Interictal epileptic discharges in the right occipital region
**P10**	15	Focal motor seizures, visual hallucination, headache	84	101	Fosphenytoin, topiramate, gabapentin, barbiturate, sodium valproate, lamotrigine (home medication), benzodiazepines (clonazepam, diazepam, midazolam, lorazepam)	Propofol, thiopental, midazolam, iv corticosteroid	N/A	SLL in the right thalamus (MRI)	Partial SE finding Severe generalized slowing
**P11**	16	Focal motor weakness, dysphasia, focal motor seizures	0	113	Levetiracetam, diazepam levetiracetam (home medication), diazepam	–	N/A	SLL in the left parietal lobe 1 month after: new SLL in the left frontal, parietal, and temporal lobes Large and confluent white matter lesions and hemosiderin (MRI)	Focal slowing in the left temporo-parietal region No epileptic discharges
	17	Confusion, headache, fatigue, focal motor weakness	0	90	Levetiracetam (home medication), diazepam		N/A	Acute SLL in the right cortical area of occipito-parietal lobe 1 month: new SLL in the left occipital lobe (MRI)	N/A
**P12**	18	Nausea, headache, visual field defect, dysphasia, confusion	0	2	No ASM	–	N/A	Normal brain MRI finding (2 weeks after the SLE)	Interictal spike-wave activity in the right temporal areas
	19	Visual field defect	0	14	Levetiracetam	–	N/A	SLL in the left occipital lobe (MRI)	Interictal spike-wave activity in the left frontal areas Mild generalized slowing
	20	Visual field defect, focal motor seizures, fatigue	0	7	Levetiracetam (previous dosage increased) lacosamide, pregabalin	–	N/A	SLL in the right occipital lobe Old cortical lesion in the left occipital lobe (MRI)	Moderate generalized slowing No epileptic discharges

Reference range for plasma lactate: 0.6–2.4 mmol/l

*ASM* anti-seizure medication, *CT* computed tomography, *EEG* electroencephalography, *ICU* intensive care unit, *MRI* magnetic resonance imaging, *N/A* no data/data not available, *NCSE* non-convulsive status epilepticus, *SE* status epilepticus, *SLE* stroke-like episode, *SLL* stroke-like lesion

Trigger events preceding SLE were noted in two patients with a *POLG* variant (P8 and P10) and with a paediatric patient with m.3243A > G (P 3). Patient 8 had fever before the onset of second SLE and Patient 10 had a dental operation. Patient 3 had influenza A tested positive at the hospital admission. In 11 cases, patients received intravenous antibiotics during the hospital stay, but the symptoms of infections were reported as having started after hospital admission. The most frequent symptoms at the onset of SLEs were focal or generalized epileptic seizures (11/20 episodes), visual symptoms (9/20), and nausea or vomiting (9/20) (Table 2). Two patients with m.3243A > G had paralytic ileus during their episode. Two patients (aged 5 and 15 at the time of SLE) received L-arginine treatment at first administration. All patients with m.3243A > G who had SLE at age over 50 years (5 patients) were diagnosed with sensorineural hearing loss before the onset of SLEs. Diabetes mellitus was also a common manifestation (4/5 patients). Two patients were diagnosed with cardiomyopathy before the first SLE (P4 and P7). Patients P6 and P1 were also diagnosed with cardiomyopathy during or after the SLE, and Patient 1 received a pacemaker due to an atrioventricular (AV) block. Three patients received medication for tachycardia during the SLE. Otherwise, electrocardiograms taken during SLEs were unremarkable. Cardiac ultrasound was not routinely performed during hospital stay. The clinical symptoms of patients at the time of first SLE are detailed in Table 1. All patients with *POLG* variants (N = 3) experienced an increased frequency of epileptic seizures before hospital admission. In these patients, visual disturbances (e.g. positive visual phenomena) and headache were also common symptoms at the onset of SLEs.

For 11 cases, plasma lactate was measured. Mean lactate was 3.0 mmol/l (range: 0.8–8 mmol/l). In the CSF samples (measured in 8/20 cases), mean lactate was 3.9 mmol/l (range: 1.9–6.9 mmol/l). Baseline plasma lactate values were available in seven patients. For these, mean value was 1.7 mmol/l (range: 1.2–2.2 mmol/l). In 14 SLE cases, a new anti-seizure medication (ASM) was started during hospital stay. Most frequent new ASMs were levetiracetam and benzodiazepines (both in 14 cases). Other commonly used ASMs were lacosamide (6 cases) and fosphenytoin (4 cases). Perampanel, carbamazepine, oxcarbazepine, topiramate, gabapentin, pregabalin and barbiturate were less commonly used (Table 2). In 11 cases, more than one ASM was used (range 1–7 anti-seizure medication, mean 3.1). Patients with *POLG* variants were treated with most ASMs. If the patient was admitted to the ICU, a general anaesthetic was used in 8/10 cases. The most common general anaesthetic was propofol (8 cases), but ketamine, thiopental, and midazolam were also used in some patients.

**Fig. 1:**
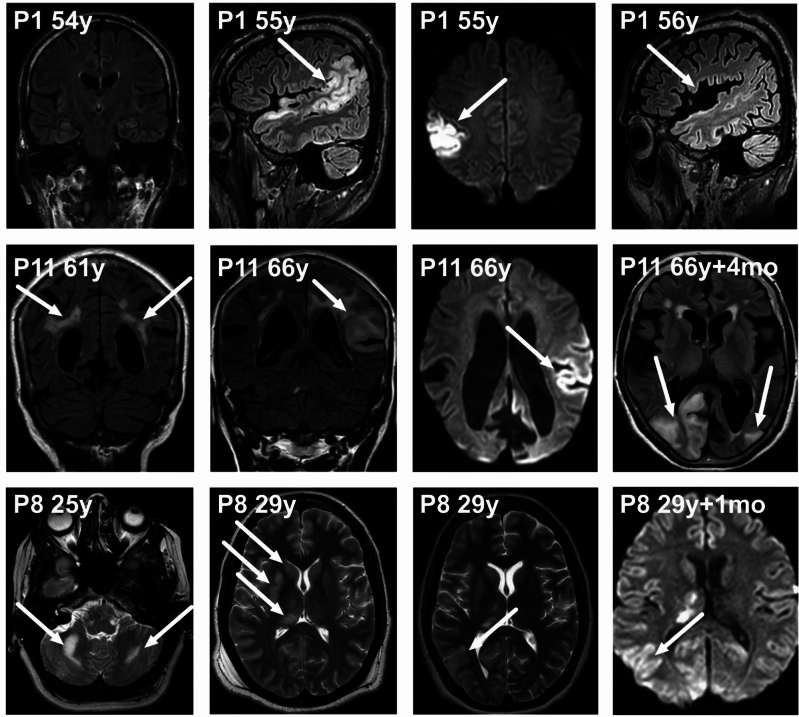
Brain MR imaging findings in patients with stroke-like episodes (SLEs)

Brain magnetic resonance (MR) imaging was performed during the SLE in 11 cases, and brain computed tomography (CT) in 17 cases. For one patient (P5, Table 1), no brain imaging data were available for three SLEs. This patient was diagnosed with m.3243A > G after the first SLE at the age of 10. The stroke-like lesions observed in brain imaging were mostly in the posterior brain regions (occipital and parietal lobes), but lesions involving the basal ganglia and thalamus were also seen in five SLEs (e.g. P8 in Fig. 1). In one patient, the stroke-like lesion extended also to the frontal lobes (P11, Table 1); this was observed in brain MR imaging obtained one month after the onset of symptoms. (Fig. 1, P11 images at 66y).

Brain MR imaging findings in patients with stroke-like episodes (SLEs). First row left to right, **P1** (see Tables 1 and 2) images: Brain coronal FLAIR at age 54 before first SLE. Sagittal FLAIR and axial Trace DWI during SLE at age 55 showing cortical oedema with restricted diffusion in the right temporo-parietal region. Repeated sagittal FLAIR at age 56 reveals atrophy in right parieto-occipital region. Second row, **P11** images: Coronal T2 at age 61 before the first SLE showing prominent white matter lesions in periventricular and subcortical areas. Coronal FLAIR and axial Trace DWI during SLE at age 66 revealing SLL in the left fronto-temporo-parietal region with restricted diffusion. Axial FLAIR four months after the patient had a new SLE at age 66, revealing SLLs in the right occipito-parietal lobe and the left occipital lobe. Third row, **P8** images: Axial T2 at age 25 before the first SLE, with inactive bilateral white matter lesions in the cerebellum. Axial T2 at age 29 during second SLE showing hyperintensities in right putamen, caudate nucleus, and the thalamus. Repeated axial T2 and axial Trace DWI one month later showing new hyperintensities in the right parietal region. P = patient. Y = years. Mo = months.

**Fig. 2:**
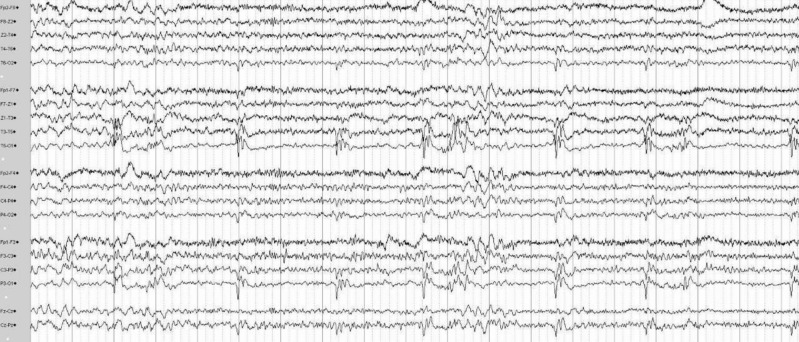
EEG during non-convulsive status epilepticus associated with *POLG* variant (patient P8)

The most common findings in the electroencephalography (EEG) recordings were mild to moderate general slowing (7 cases) and epileptic discharges (8 cases), most commonly in the posterior regions (Table 2). Two patients with *POLG* variants (both at age < 30 years) had super refractory status epilepticus. Both were treated for long periods (33 and 84 days) in the ICU and multiple ASMs were used (Table 2). In addition to ASMs, a ketogenic diet was used in both patients and intravenous corticosteroids in one. Both patients ultimately died from the SLE, one 103 days and the other 193 days after the onset of SLE symptoms. Figure 2 shows the EEG findings obtained during a non-convulsive status epilepticus of patient P8. This patient has been reported previously [13].

The twenty-three-channel EEG shows continuous, quasi-rhythmic polyspike-and-delta wave activity within the left occipital region (maximum at O1 electrode). Recording was performed with NicoletOne EEG (Nervus device, Cephalon Ltd., Nørresundby, Denmark), Electrocap with Ag–AgCl-electrodes, and standard international 10/20 electrode placement. The space between each vertical line represents one second.

In 12 SLE cases, the patients needed prolonged hospital treatment. Only in five episodes were patients discharged directly home from TUH. Based on the available medical record data, the median modified Rankin scale [14] (mRS) value before the episode was mRS = 2 (range 0–4) and mRS = 4 (range 0–6) three months after the SLE.

## Discussion

In this study, we report the clinical features, treatment, and outcomes of 20 stroke-like episodes in 12 patients. The mean age at first SLE was 40 years, 42 years in the m.3243A > G subgroup. The presence of developmental problems has been described before as a sign of earlier onset of SLE in m.3243A > G patients [15]. This was also seen in our study (P3 and P5). The phenomenon of late-onset SLE has been increasingly recognized. In a large UK study, the mean age at first SLE was 31.8 years and 35.2 years in the mtDNA subgroup [6]. Other studies on SLEs have reported a mean age of 27 years with m.3242A > G carriers [15], and a median age of 45 years in patients with the MELAS phenotype [16]. In our cohort the mean age was older, 56 years at first SLE in adult patients with m.3243A > G. These results underline the importance of considering the possibility of mitochondrial SLE also in older individuals when they develop new subacute or acute neurological symptoms and findings compatible with an SLE.

All the patients who presented with their first SLE at the age of > 50 years had some mitochondrial disease symptoms in their clinical history (Table 1). However, two patients were diagnosed with mitochondrial disease only after their first SLE. The suspicion of SLE arose from the clinical symptoms and brain imaging findings, with low BMI and short stature being additional suggestive factors. Sensorineural hearing loss, short stature, and low BMI have been identified as risk factors for more severe disease burden in m.3242A > G patients in previous studies [6, 15–18].

Trigger events preceding SLEs were noted in three cases at the hospital admission. In a study on paediatric SLE, 36% of the cases, with variable genetic aetiologies, had an infection as a trigger event [19]. In our data, this association was more clearly seen in patients with *POLG* variants. Indeed, recent studies suggest that an abnormal innate immune response can trigger the acute epileptic form of the POLG disease [20].

Our study included three patients with *POLG* variants and stroke-like episodes. *POLG* variants are known to cause stroke-like episodes [7, 8] and the features in SLE seem to be similar in patients with mtDNA and *POLG* variants. However, in *POLG* associated mitochondrial disease, the SLEs start later than other neurological features and motor seizures and status epilepticus are more common [6]. In two patients (P8 and P10, Table 2) visual symptoms preceded epileptic symptoms in SLE and this has been reported in previous studies with *POLG* variants [21, 22].These could be considered as initial symptoms of SLE. The distinction from prodromal symptoms to SLE symptoms is challenging due to the evolving nature of episodes. The stroke-like lesions in *POLG* variants are commonly located in posterior brain regions, especially in the occipital lobe [23], but also thalamic lesions have been reported [6, 8, 24]. In patients P8 and P10, thalamic lesions were also observed during the SLEs (P8, Fig. 1). The mean onset age of *POLG*-related seizures and stroke-like episodes has been reported to be 17–18 years, which is significantly lower than patients with mtDNA variants [6, 8]. This difference was also observed in our study with patients P8 and P10, who both presented with their first SLE in their twenties. Patient P9 harboured the homozygous variant W748S in *POLG* and experienced his first SLE at the age of 61 years, but other neurological symptoms started before the age of 40.

Epilepsy is a common manifestation in both mtDNA and nDNA associated mitochondrial disorders. Prevalence of seizures has been reported as 34.9% with the m.3243A > G genotype and 92.3% with the m.8344A > G [25]. Altogether 128 pathogenic *POLG* variants have been associated with epilepsy, W748S, A467T and G848S being the most common variants [8]. In our study, all patients with *POLG* disease harboured the W748S homozygous variant. Only two patients (P9 and P10, both with *POLG* variants) were diagnosed with epilepsy before their first SLE. In this study, 8/12 patients were diagnosed with epilepsy only after first or second SLE and ASM was initiated. Focal seizures, accompanied with headache and vomiting, as well as epileptiform discharges with predilection in occipital regions are most commonly reported in *POLG* associated epilepsy [21, 24, 26]. This was also observed in our study. Patient P12 harboured the m.8344A > G mutation and was diagnosed with epilepsy at the age of 25. He had more severe epilepsy than other patients with mtDNA mutation. In patients harbouring the m.8344A > G mutation, epilepsy is typically of earlier onset than in patients with the m.3243A > G [25].

There are no randomized trial data on seizure management in patients with mitochondrial disease. The treatment of mitochondrial epilepsy associated with *POLG* variants is particularly challenging as super-refractory status epilepticus is not uncommon. The consensus-based recommendation from 2019 advices prompt initiation of intravenously administered ASM [9] and the general consensus is to avoid ASMs with known mitochondrial toxicity, especially valproic acid [9]. The recently published consensus statement for managing seizures in patients with primary mitochondrial disease recommends following the National Institute for Health and Care Excellence (NICE) general guidelines for seizure management, but advices against valproic acid in POLG patients, against vigabatrin in γ-aminobutyric acid transaminase deficiency, and against topiramate in patients at risk for renal tubular acidosis [27].

A recent study consisting 19 *POLG* patients recommends a combination of sodium channel blocker and benzodiazepine for generalized tonic–clonic seizures, and topiramate, phenobarbital or clonazepam for myoclonus [21]. In our study, the most commonly used ASM was levetiracetam, which is commonly used in mitochondrial epilepsy and has reported to be both safe and efficient [28, 29]. Propofol was the most used general anaesthetic in our study. Propofol should be used with care because of the potential risk for propofol infusion syndrome [9]. None among our patients developed propofol infusion syndrome. The typical reason to start other general anaesthetics than propofol was the insufficient effect of propofol in super-refractory status epilepticus. In our data, one patient was administered sodium valproate against seizures and developed ultimately fatal hepatic failure. This patient was diagnosed only *post mortem* with *POLG* disease, and has been reported earlier in a case series of patients with *POLG* epilepsy [22]. During that time, the risks of sodium valproate in patients with possible *POLG* epilepsy were not yet well appreciated [30]. Two of our patients were given L-arginine as SLE treatment. Both were under the age of 16 years at the time of first administration. However it should be noted that the evidence regarding the safety and efficacy of L-arginine as a treatment of SLEs remains scarce [31], and there is no expert consensus to endorse its use [9].

The typical subacute onset of SLEs was evident in our study [9, 16]. The duration of hospital stay during the SLE acute phase has not been widely reported earlier. In our study, the large range in the hospital stay duration and outcome variability are evident. These may create challenges in clinical decision making during prolonged acute episodes, and better prognostic tools are needed. We also evaluated the mRS score before and 3 months after hospital discharge. Despite the often-reversible nature of SLEs, many patients reported new symptoms affecting their daily living after the episode, suggesting that non-reversible disease progression associated with SLEs might merit further study. MRS scores were retrospectively evaluated from the medical records of the patients, which is a potential limitation.

Stroke-like episodes are associated with risk of early death in both patients with m.3243 > G and *POLG* variants [6]. In *POLG* patients also earlier disease onset, previous diagnosis of epilepsy, and compound heterozygous variants are associated with worse prognosis [12]. Median age at death was 25 years in a cohort of 33 patients with homozygous W748S variant in *POLG* [8]. In our three *POLG* patients, who all harboured the same W748S variant, the median age at death was 30 years. The median survival time in *POLG* patients from seizure onset to death was 34 months in our cohort, which is quite similar (37 months) to that described in another recent study [12]. In patients with m.3243A > G the mean age at death is described being higher at 45.8 years [6]. Among all deceased patients with m.3243A > G in our study (three patients, among whom two had a history of SLE) the mean age at death was 63 years. One reason for longer survival may be the later onset of SLEs among the patients with m.3243A > G in our study.

Detailed information on the clinical course, treatment, and outcomes related to SLEs are needed to improve early recognition and treatment of this potentially life-threatening entity. The prompt management of the symptoms, especially epileptic seizures, is of importance as many of the patients needed ICU treatment for status epilepticus. It is also of crucial importance to share information about SLEs with patients with mitochondrial disease who are at high risk for SLE, as well as their close ones. In our study, only a few patients were in contact with healthcare workers at the onset of symptoms. Most commonly, the symptoms had already evolved over a few days before the administration to hospital, and this delay can partly explain the poor outcomes in some of the patients. In addition, efforts should also be directed at increasing the awareness of SLEs in primary care, where patients may turn for advice when they experience new symptoms.
